# Cerebral small vessel disease as imaging biomarker predicting ocular cranial nerve palsy of presumed ischemic origin at admission

**DOI:** 10.1038/s41598-022-16413-x

**Published:** 2022-07-18

**Authors:** Dong-Wan Kang, Sue Young Ha, Jung-Joon Sung, Hyunwoo Nam

**Affiliations:** 1 Department of Neurology, Seoul National University Hospital and Seoul National University College of Medicine, 101, Daehak-ro, Jongno-gu, Seoul, 03080 Republic of Korea; 2 Department of Neurology, Seoul Metropolitan Government-Seoul National University Boramae Medical Center and Seoul National University College of Medicine, 20, Boramae-ro 5-gil, Dongjak-gu, Seoul, 07061 Republic of Korea

**Keywords:** Neurological disorders, Diagnostic markers, Predictive markers

## Abstract

Ocular cranial nerve palsy of presumed ischemic origin (OCNPi) is the most common type of ocular cranial nerve palsy (OCNP) in patients aged ≥ 50 years; however, no definite diagnostic test exists. As diagnostic criteria include clinical improvement, diagnoses are often delayed. Diagnostic biomarkers for OCNPi are required; we hypothesized that cerebral small vessel disease is associated with OCNPi. We analyzed 646 consecutive patients aged ≥ 50 years with isolated unilateral OCNP who underwent work-ups at two referral hospitals. White matter hyperintensities (WMHs), silent infarctions, and cerebral microbleeds (CMBs) were assessed. In multivariate analyses, mild (grades 1–3) and moderate to severe (grades 4–6) WMHs were significantly associated with OCNPi compared to OCNP of other origins (odds ratio [OR] 3.51, 95% confidence interval [CI] 1.91–6.43, *P* < 0.001; OR 3.47, 95% CI 1.42–8.48, *P* = 0.006, respectively). Silent infarction and CMBs did not remain associated (OR 0.96, 95% CI 0.54–1.70, *P* = 0.870; OR 0.55, 95% CI 0.28–1.08, *P* = 0.080, respectively). Associations between WMH and OCNPi remained after excluding patients with vascular risk factors. In conclusion, the presence of WMH could independently predict ischemic origin in patients with isolated unilateral OCNP at early stage of admission.

## Introduction

Ocular cranial nerve palsy (OCNP) is a neurological deficit that occurs due to dysfunction of one or more oculomotor, trochlear, and abducens nerves. It is important to clarify the cause of OCNP because the management plan and prognosis will be completely different depending on its cause. The possible causes of OCNP include microvascular ischemia, tumor, infection, aneurysm, trauma, and vascular malformation^1^. Careful history-taking, physical examination, and neuroimaging studies are helpful in revealing the causes of OCNP^2^.

OCNP of presumed ischemic origin (OCNPi) is the most common cause of OCNP among patients aged ≥ 50 years^3, 4^. However, it has no definitive diagnostic test, and other possible causes should be ruled out before diagnosis^5^. Vascular risk factors, such as diabetes, hypertension, and hyperlipidemia, could increase the probability of OCNPi, but it can occur in patients without any history of vascular risk factors^5^. OCNPi can be diagnosed when (i) no other cause is found on clinical examination or neuroimaging, (ii) there is no other accompanying neurological deficit, and (iii) symptoms improve over 3–6 months^6, 7^. In other words, a confirmatory diagnosis cannot be made until symptoms improve. This uncertainty in diagnosis could worry patients during the follow-up. Magnetic resonance imaging (MRI) findings predicting OCNPi could help reduce this.

Ischemic OCNP occurs due to endothelial deformation, loss of tight junctions, basement membrane hypertrophy, and loss of pericytes, leading to endoneurial space edema and hypoxia in ocular cranial nerves^8^. Arteriolar hyalinization has been observed in post-mortem studies of OCNPi patients^9^. In brains of patients with vascular risk factors, the arteriolar and capillary levels of the vasculature can be affected, resulting in cerebral small vessel disease (SVD)^10^. SVD is well visualized on MRI and is characterized by heterogeneous findings, such as white matter hyperintensities (WMH), silent infarction, cerebral microbleeds (CMBs), and enlarged perivascular space^10^. Thus, we hypothesized that cerebral SVD could be associated with OCNPi. In this study, the imaging markers of cerebral SVD, WMHs, silent infarction, and CMBs on MRI of patients with acute-onset isolated OCNP were evaluated.

## Methods

### Study population, inclusion criteria, and exclusion criteria

We retrospectively reviewed the electronic and paper medical records of consecutive patients aged ≥ 50 years with isolated oculomotor, trochlear, or abducens nerve palsy diagnosed by neurologists and were admitted to the Seoul National University Hospital and the Seoul National University-Seoul Metropolitan Government Boramae Medical Center, South Korea between January 2000 and April 2020. We collected eligible cases using the following inclusion criteria: (i) isolated unilateral OCNP, (ii) OCNP unrelated to trauma or neurosurgery, (iii) no previous history of strabismus, and (iv) diagnostic workup including MRI. Isolated unilateral OCNP was defined as unilateral oculomotor, trochlear, or abducens nerve palsy with no other neurological symptoms or signs, except for headache and periorbital pain. The exclusion criteria were as follows: (i) poor MRI quality; (ii) incomplete work-up to support a final diagnosis; and (iii) follow-up loss within 6 months and inability to confirm symptom improvement. Because this study aimed to investigate OCNP induced by ischemia of the vasa nervorum, OCNP cases caused by stroke, which is a brain parenchymal ischemia due to occlusion of other intracranial arteries, were excluded. The study protocol was designed in accordance with the Declaration of Helsinki. The Institutional Review Boards (IRB) of the Seoul National University Hospital and Seoul National University-Seoul Metropolitan Government Boramae Medical Center approved this study (IRB No. J-2007-023-1139 and 10-2019-39, respectively) and waived the requirement to obtain informed consent because of its retrospective design.

### Collection of clinical information

We collected the participants’ baseline demographic and clinical information, including age, sex, body mass index (BMI), hypertension, diabetes, hyperlipidemia, chronic kidney disease, history of ischemic heart disease, history of stroke, current cigarette smoking, and use of antithrombotics. Laboratory findings of hemoglobin A1c (HbA1c) and estimated glomerular filtration rate (eGFR) were collected.

The affected nerve, cause of OCNP, and presence of periorbital pain or headache were reviewed. OCNPi was diagnosed when symptoms spontaneously improved within 12 months of the follow-up period, with no other explainable diagnoses. Patients with no symptom improvement for > 1 year of follow-up and no explainable diagnosis were considered idiopathic.

### Image analysis

The patients were examined using either a 3 T or 1.5 T MRI unit. The types of scanner and imaging acquisition parameters are summarized in Supplementary Table S1 and Supplementary Fig. S1. For WMH, each periventricular WMH (PVWMH) and deep WMH (DWMH) was graded as previously described. PVWMH was graded as Grade 0 (absence), 1 (caps or pencil-thin lining), 2 (smooth halo), or 3 (irregular PVWMH extending into the deep white matter). DWMH was graded as 0 (absence), 1 (punctate foci), 2 (beginning confluence of foci), or 3 (large confluent areas)^11^. Total WMH was graded by summing the PVWMH and DWMH, which ranged from 0 to 6. The total WMH was divided into three groups as follows: no WMH, grade 0; mild WMH, grades 1–3; and moderate to severe WMH, grades 4–6^12–14^. We defined silent infarction as abnormal low-signal intensity lesions of at least 3 mm in size on fluid-attenuated inversion recovery (FLAIR) with or without hyperintense rim^15^. CMBs were defined as dark blooming artifacts visible on T2*-weighted gradient echo (GRE) or susceptibility weighted images (SWI) surrounded by the brain parenchyma^15^. Two independent examiners evaluated MRI images without clinical information. The inter-rater agreement for the imaging parameters was assessed using the intraclass correlation coefficient (ICC) and 95% confidence interval (CI).

### Statistical analysis

In the univariate analysis, Student’s *t*-tests and chi-square tests were used for continuous and categorical variables, respectively. Multivariate logistic regression analysis models were constructed using imaging markers as dependent variables with adjustment for relevant confounding variables. We also considered the MRI field strength (3 T vs. 1.5 T) and susceptibility sequence type (SWI vs. GRE) as possible confounding variables of the WMH and silent infarction and CMBs, respectively. Statistical significance was set at *P* < 0.05. All statistical analyses were performed using R (version 4.0.2; R Development Core Team, Vienna, Austria).

## Results

**Figure 1:**
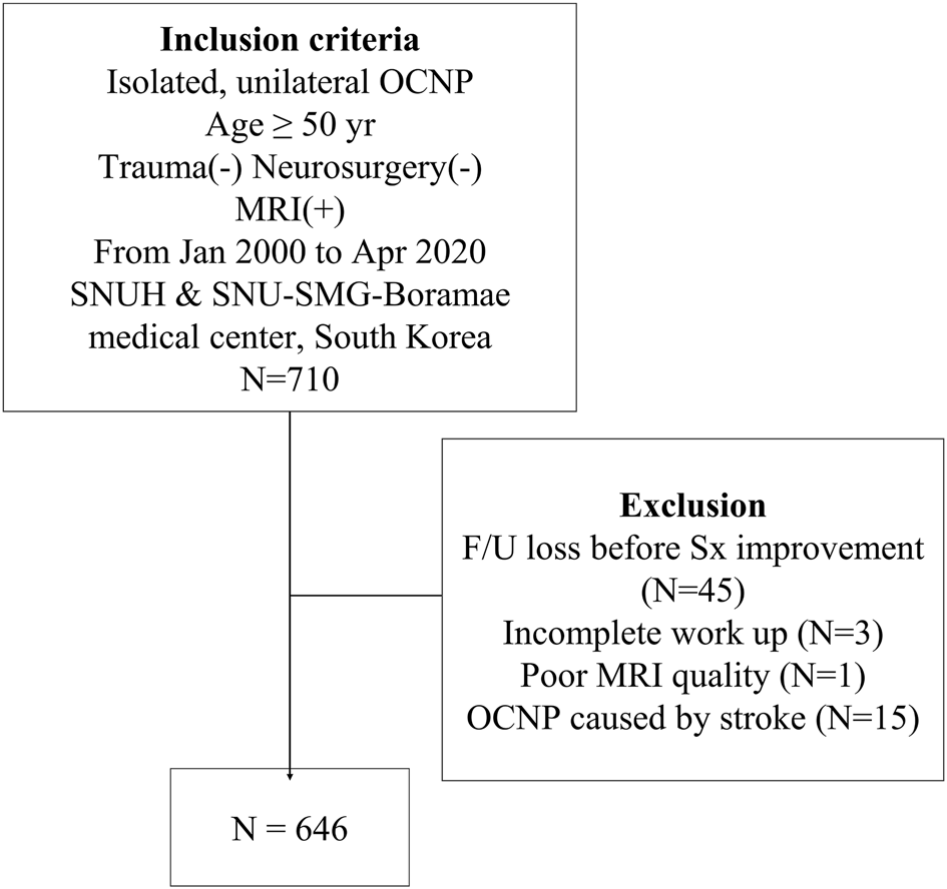
Flow chart of patient enrollment.

We recruited 710 patients with acute-onset isolated unilateral OCNP who underwent diagnostic workup, including MRI, from January 2000 to April 2020. After excluding 64 cases, 646 were analyzed (61.6% male, mean age 67 ± 9 years) (Fig. 1). The baseline characteristics of the patients are shown in Table 1 and Supplementary Table S2. The proportions of oculomotor, trochlear, and abducens nerves were 39.5%, 21.5%, and 39.0%, respectively. The proportion of patients with OCNPi was 73.2% (473 patients). The detailed causes of OCNP and their respective proportions are listed in Table 2 and Supplementary Table S3. Three-tesla and 1.5 T MRI were used in 298 (46.1%) and 348 (53.9%), respectively, and SWI and GRE sequences were used in 244 (37.8%) and 327 (50.6%), respectively. Of the suggested MRI acquisition standards for neuroimaging for clinical research^16^, all MRIs met the in-plane resolution criteria of ≤ 1 mm × 1 mm, and 97.5% of MRIs met the slice thickness criteria of ≤ 5 mm. WMH, PVWMH, and DWMH were present in 512 (79.3%), 374 (57.9%), and 477 (73.8%) patients, respectively. Silent infarctions and CMBs were visualized in 152 (23.6%) and 86 (15.1%) cases, respectively. The inter-rater agreement of WMH, silent infarction, and CMBs was substantial for MRI parameters (Cohen’s kappa values of 0.78, 0.79, and 0.88, respectively; *P* < 0.001 for all parameters).

**Table 1:** Baseline characteristics of the included patients. *OCNPi* ocular cranial nerve palsy of presumed ischemic origin, *BMI* body mass index, *CN* cranial nerve, *OCNP* ocular cranial nerve palsy, *WMH* white matter hyperintensities, *PVWMH* periventricular white matter hyperintensities, *DWMH* deep white matter hyperintensities, *CMBs* cerebral microbleeds, *GRE* T2*-weighted gradient echo, *SWI* susceptibility weighted image, *IHD* ischemic heart disease, *eGFR* estimated glomerular filtration rate, *OCNP* ocular cranial nerve palsy. Vascular risk factor refers to the presence of at least one of the following: hypertension, diabetes, dyslipidemia, and/or smoking.

	Total (N = 646)
**Male sex**	398 (61.6%)
**Age**	67 ± 9
**BMI (kg/m**^**2**^**)**	24.4 ± 3.5
**Diagnosis**	
CN III	255 (39.5%)
CN IV	139 (21.5%)
CN VI	252 (39.0%)
**OCNPi**	473 (73.2%)
**Periorbital pain or headache**	284 (44.0%)
**WMH**	512 (79.3%)
No (0)	134 (20.7%)
Mild (1–3)	409 (63.3%)
Moderate to severe (4–6)	103 (15.9%)
**PVWMH**	374 (57.9%)
No (0)	272 (42.1%)
Mild (1)	219 (33.9%)
Moderate to severe (2–3)	155 (24.0%)
**DWMH**	477 (73.8%)
No (0)	169 (26.2%)
Mild (1)	387 (59.9%)
Moderate to severe (2–3)	90 (13.9%)
**Silent infarction**	152 (23.6%)
**CMBs**	86 (15.1%)
**MRI field strength**	
1.5 T	348 (53.9%)
3 T	298 (46.1%)
**Susceptibility sequence**	
GRE	327 (50.6%)
SWI	244 (37.8%)
**Vascular risk factors**	502 (77.7%)
**Hypertension**	341 (52.8%)
**Diabetes**	276 (42.7%)
**Dyslipidemia**	174 (26.9%)
**Smoking**	98 (15.2%)
**IHD**	62 (9.6%)
**History of stroke**	33 (5.1%)
**HbA1c (%)**	6.7 ± 1.5
**Chronic kidney disease**	80 (12.4%)
**eGFR (mL/min/1.73 m**^**2**^**)**	82.0 ± 24.9
**Prior use of antithrombotics**	126 (19.6%)
**Follow up duration after OCNP (yr)**	4.8 ± 3.9

**Table 2:** Causes of ocular cranial nerve palsy (OCNP). *P-com* posterior communicating artery, *AchA* anterior choroidal artery, *dICA* distal internal carotid artery, *dAVF* dural arteriovenous fistula.

Etiology	Disease
Presumed ischemic origin (473, 73.2%)	Presumed ischemic origin (473)
Aneurysm (31, 4.8%)	P-com aneurysm (22) AchA aneurysm (1) dICA aneurysm (8)
Cavernous sinus vascular lesions (21, 3.3%)	Cavernous sinus thrombosis (1) Cavernous-carotid fistula (5) cavernous dAVF (15)
Infection (5, 0.8%)	Skull base osteomyelitis (2) Herpes zoster ophthalmicus (1) Invasive fungal sinusitis (1) Sphenoid sinusitis (1)
Metabolic disorders (1, 0.2%)	Wernicke encephalopathy (1)
Inflammation (35, 5.4%)	Multiple sclerosis (1) Inflammatory pseudotumor or Tolosa-Hunt syndrome (29) Vasculitis (3) Pachymeningitis (1) Wegener’s granulomatosis (1)
Autoimmune (35, 5.4%)	Miller–Fisher syndrome (6) Myasthenia gravis (23) Thyroid ophthalmopathy (6)
Neoplasm/malignancy (39, 6.0%)	Pituitary tumor (7) Meningioma (6) Lymphoma (5) Leptomeningeal seeding (5) Brain metastasis (6) Nasopharyngeal carcinoma (4) Mucocele (2) Multiple myeloma (2) Posterior fossa epidermoid cyst (1) Bone metastasis (1)
Others (6, 0.9%)	Spontaneous intracranial hypotension (1) Idiopathic (5)

We analyzed factors associated with OCNPi compared to OCNP of other origin (OCNPo) using univariate analyses (Table 3). Of the 473 patients with OCNPi, 393 (83.1%) had WMH, and 119 (68.8%) of the 173 patients with OCNPo had WMH (*P* < 0.001). Both mild (grades 1–3) and moderate to severe (grades 4–6) WMH were more frequent in patients with OCNPi than in those with OCNPo. Both PVWMH and DWMH were also associated with OCNPi (PVWMH, 292 [61.7%] vs. 82 [47.4%]; DWMH, 363 [76.7%] vs. 114 [65.9%] in ischemic and OCNPo, respectively; both *P* = 0.007). Silent infarction was more prevalent in patients with OCNPi than those with OCNPo (OCNPi vs. the OCNPo, 122 [25.8%] vs. 30 [17.3%], *P* = 0.032). No association was observed between CMBs and OCNPi. Although the proportion of mild PVWMH was higher on 3 T MRI than on 1.5 T MRI, the proportion of WMH was comparable between 3 T and 1.5 T MRI (Supplementary Table S4). CMBs were more frequently visualized using SWI than using GRE (Supplementary Table S5). The proportions of 3 T MRI (vs. 1.5 T MRI) and SWI (vs. GRE) were also comparable between OCNPi and OCNPo. Among the clinical variables, male sex, old age, high BMI, hypertension, diabetes, history of ischemic heart disease, eGFR, and antithrombotic use were associated with OCNPi. The presence of at least one vascular risk factor among hypertension, diabetes, hyperlipidemia, and smoking was 83.9% (397/473) for OCNPi and 60.7% (105/173) for OCNPo (*P* < 0.001).

**Table 3:** Factors associated with ocular cranial nerve palsy of presumed ischemic origin (OCNPi). *BMI* body mass index, *CN* cranial nerve, *OCNP* ocular cranial nerve palsy, *WMH* white matter hyperintensities, *PVWMH* periventricular white matter hyperintensities, *DWMH* deep white matter hyperintensities, *CMBs* cerebral microbleeds, *GRE* T2*-weighted gradient echo, *SWI* susceptibility weighted image, *IHD* ischemic heart disease, *eGFR* estimated glomerular filtration rate. Vascular risk factor refers to the presence of at least one of the following: hypertension, diabetes, dyslipidemia, and/or smoking. *Student’s *t* tests for continuous variables and Chi-square tests for categorical variables.

	OCNPi (N = 473)	OCNPo (N = 173)	*P*-value*
**Male sex**	318 (67.2%)	80 (46.2%)	< 0.001
**Age**	67.3 ± 8.4	65.1 ± 9.3	0.004
**BMI (kg/m**^**2**^**)**	24.7 ± 3.3	23.8 ± 3.9	0.025
**Diagnosis**			< 0.001
CN III	159 (33.6%)	96 (55.5%)	
CN IV	123 (26.0%)	16 (9.2%)	
CN VI	191 (40.4%)	61 (35.3%)	
**Periorbital pain or headache**	199 (42.1%)	85 (49.1%)	0.131
**WMH**	393 (83.1%)	119 (68.8%)	< 0.001
No (0)	80 (16.9%)	54 (31.2%)	< 0.001
Mild (1–3)	306 (64.7%)	103 (59.5%)	
Moderate to Severe (4–6)	87 (18.4%)	16 (9.2%)	
**PVWMH**	292 (61.7%)	82 (47.4%)	0.001
No (0)	181 (38.3%)	91 (52.6%)	0.001
Mild (1)	164 (34.7%)	55 (31.8%)	
Moderate to severe (2–3)	128 (27.1%)	27 (15.6%)	
**DWMH**	363 (76.7%)	114 (65.9%)	0.007
No (0)	110 (23.3%)	59 (34.1%)	0.007
Mild (1)	289 (61.1%)	98 (56.6%)	
Moderate to severe (2–3)	74 (15.6%)	16 (9.2%)	
**Silent infarction**	122 (25.8%)	30 (17.3%)	0.032
**CMBs**	62 (14.9%)	24 (15.8%)	0.889
**MRI field strength**			0.403
1.5 T	260 (55.0%)	88 (50.9%)	
3 T	213 (45.0%)	85 (49.1%)	
**Susceptibility sequence**			0.720
GRE	237 (56.7%)	90 (58.8%)	
SWI	181 (43.3%)	63 (41.2%)	
**Vascular risk factors**	397 (83.9%)	105 (60.7%)	< 0.001
**Hypertension**	271 (57.3%)	70 (40.5%)	< 0.001
**Diabetes**	248 (52.4%)	28 (16.2%)	< 0.001
**Dyslipidemia**	136 (28.8%)	38 (22.0%)	0.105
**Smoking**	75 (15.9%)	23 (13.3%)	0.497
**IHD**	53 (11.2%)	9 (5.2%)	0.032
**History of stroke**	29 (6.1%)	4 (2.3%)	0.08
**HbA1c (%)**	6.9 ± 1.6	6.2 ± 1.2	< 0.001
**Chronic kidney disease**	65 (13.8%)	15 (8.7%)	0.108
**eGFR (mL/min/1.73 m**^2^)	80.2 ± 23.7	86.9 ± 27.2	0.005
**Prior use of antithrombotics**	106 (22.5%)	20 (11.6%)	0.003
**Follow up duration after OCNP (yr)**	4.8 ± 4.0	4.7 ± 3.9	0.749

**Figure 2:**
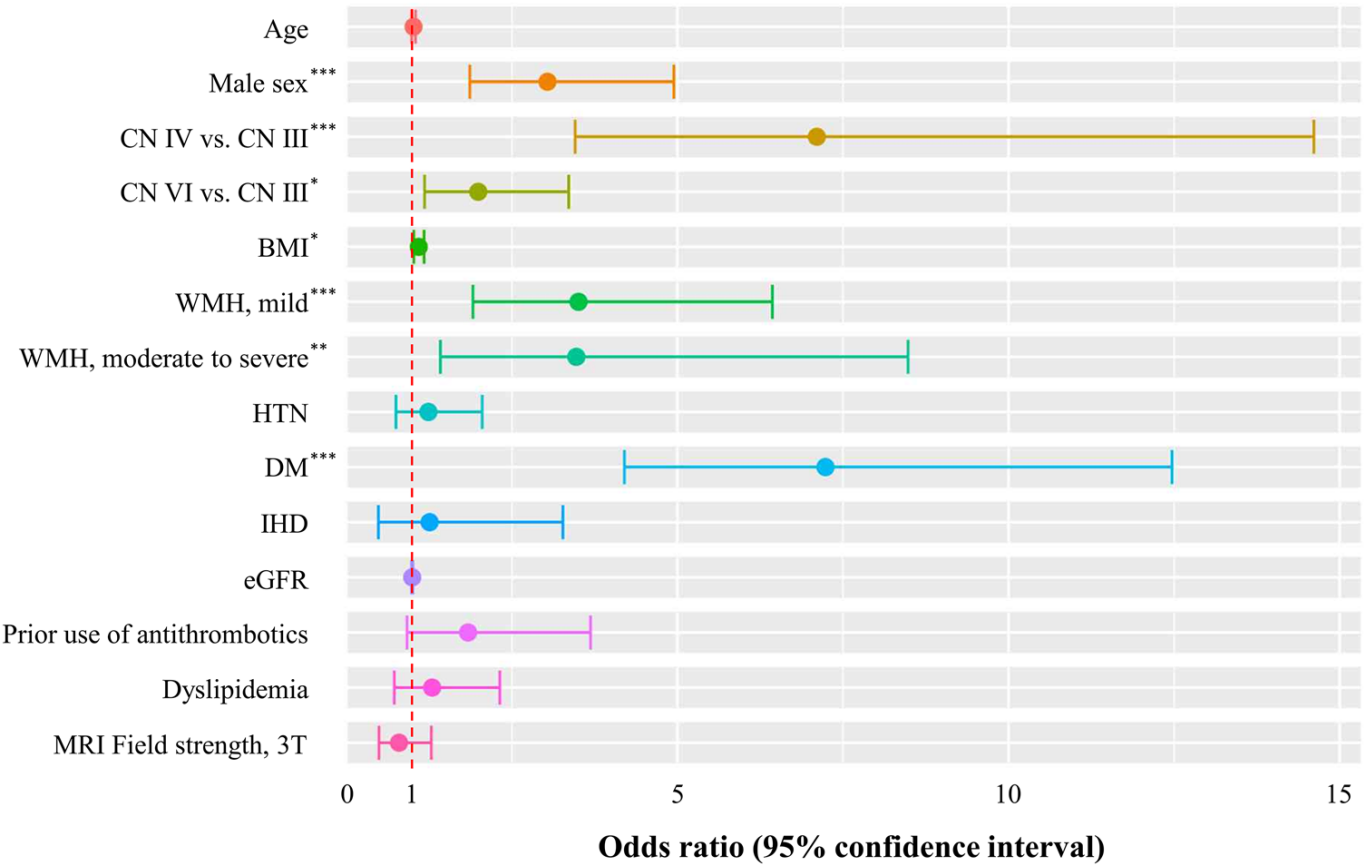
Forest plots for odds ratios of the ocular cranial nerve palsy of presumed ischemic origin (OCNPi) compared with ocular cranial nerve palsy of other origin (OCNPo) based on multivariate logistic regression analysis. *CN* cranial nerve, *BMI* body mass index, *WMH* white matter hyperintensities, *HTN* hypertension, *DM* diabetes, *IHD* ischemic heart disease, *eGFR* estimated glomerular filtration rate. ***P < 0.001; **P < 0.01; *P < 0.05.

In the multivariate analysis, both mild (grades 1–3) and moderate to severe (grades 4–6) WMH were independent predictors of OCNPi (Table 4 and Fig. 2). The odds ratio (OR) and 95% CI of mild and moderate-to-severe WMH were 3.51 [1.91–6.43] and 3.47 [1.42–8.48], respectively (*P* < 0.001 and *P* < 0.006, respectively). Among the covariates, trochlear and abducens nerve involvement, male sex, high BMI, and diabetes were independent variables associated with OCNPi. Multivariate analyses were performed to investigate whether each MRI marker, silent infarction, and CMBs were independently associated with OCNPi (Table 5). There was no clear association between OCNPi and CMBs or silent infarctions. Neither PVWMH nor DWMH alone showed any association with the OCNPi (Supplementary Table S6).

**Table 4:** Predictors of ocular cranial nerve palsy of presumed ischemic origin (OCNPi) based on multivariate logistic regression analysis (Model 1). *CN* cranial nerve, *BMI* body mass index, *WMH* white matter hyperintensities, *IHD* ischemic heart disease, *eGFR* estimated glomerular filtration rate, *CI* confidence interval.

	OCNPi	
	Odds ratio (95% CI)	*P*-value
**Age**	1.02 (0.99–1.05)	0.303
**Male sex**	3.04 (1.87–4.95)	< 0.001
**Cranial nerve**		
CN III	Reference	Reference
CN IV	7.11 (3.46–14.61)	< 0.001
CN VI	1.99 (1.18–3.36)	0.010
**BMI**	1.09 (1.02–1.18)	0.012
**WMH**		
No (0)	Reference	Reference
Mild (1–3)	3.51 (1.91–6.43)	< 0.001
Moderate to Severe (4–6)	3.47 (1.42–8.48)	0.006
**Hypertension**	1.24 (0.75–2.05)	0.402
**Diabetes**	7.24 (4.20–12.47)	< 0.001
**IHD**	1.26 (0.48–3.27)	0.637
**eGFR**	1.00 (0.99–1.01)	0.386
**Prior use of antithrombotics**	1.84 (0.92–3.69)	0.086
**Dyslipidemia**	1.3 (0.73–2.32)	0.381
**MRI Field strength, 3 T**	0.8 (0.49–1.28)	0.350

**Table 5:** Odds ratios of the imaging parameters based on multivariate logistic regression analysis. In Model 2, covariates were age, sex, diagnosis, susceptibility sequence type [susceptibility weighted image (SWI) vs. T2*-weighted gradient echo (GRE)], body mass index (BMI), hypertension, diabetes, ischemic heart disease (IHD), estimated glomerular filtration rate (eGFR), prior use of antithrombotics, and dyslipidemia. In Model 3, covariates were age, sex, diagnosis, MRI field strength (3 T vs. 1.5 T), BMI, hypertension, diabetes, IHD, eGFR, prior use of antithrombotics, and dyslipidemia. *OCNPi* ocular cranial nerve palsy of presumed ischemic origin, *CMBs* cerebral microbleeds, *CI* confidence interval.

	OCNPi	
	Odds ratio (95% CI)	*P*-value
**Model 2. CMBs**	0.55 (0.28–1.08)	0.080
**Model 3. Silent infarction**	0.95 (0.54–1.70)	0.870

To exclude the confounding effects of vascular risk factors, we performed sensitivity analysis in patients without any vascular risk factors. A total of 144 patients were analyzed, and the baseline characteristics and factors associated with OCNPi are described in Supplementary Table S7 (76 patients with OCNPi vs. 68 patients with OCNPo). ORs for mild (grades 1–3) and moderate to severe (grades 4–6) WMH and OCNPi were 4.79 (95% CI 1.23–18.65, *P* = 0.024) and 11.58 (95% CI 1.29–104.18, *P* = 0.029), respectively (Table 6). There was no clear association between other SVD biomarkers and OCNPi.

**Table 6:** A sensitivity analysis among patients without any of vascular risk factors (n = 144). Odds ratios of the imaging parameters were represented based on multivariate logistic regression analysis. In Models A and C, covariates were age, sex, diagnosis, body mass index (BMI), ischemic heart disease (IHD), estimated glomerular filtration rate (eGFR), prior use of antithrombotics, and MRI field strength (3 T vs. 1.5 T). In Model B, covariates were age, sex, diagnosis, BMI, IHD, eGFR, prior use of antithrombotics, and susceptibility sequence type [susceptibility weighted image (SWI) vs. T2*-weighted gradient echo (GRE)]. *OCNPi* ocular cranial nerve palsy of presumed ischemic origin, *WMH* white matter hyperintensities, *CMBs* cerebral microbleeds.

	OCNPi	
	Odds ratio (95% CI)	*P*-value
**Model A. WMH**		
No (0)	Reference	Reference
Mild (1–3)	4.79 (1.23–18.65)	0.024
Moderate to severe (4–6)	11.58 (1.29–104.18)	0.029
**Model B. CMBs**	1.10 (0.25–4.85)	0.896
**Model C. Silent infarction**	0.91 (0.23–3.59)	0.891

## Discussion

The clinical characteristics of 646 patients aged ≥ 50 years who were diagnosed with acute-onset unilateral isolated OCNP were investigated. We enrolled patients aged ≥ 50 years for this study because OCNPi is uncommon in younger patients^17^, and extrinsic causes such as trauma-related and surgery-related OCNP were not included to focus on the intrinsic factors associated with OCNPi. Because this study aimed to investigate ischemia of the peripheral cranial nerves, ischemia of the nuclear or fascicular lesions of the ocular cranial nerve caused by stroke were excluded. In this study, mild and moderate-to-severe WMHs were found to be independent predictors of OCNPi.

The positive finding we found on early MRI is meaningful as it might be used as a predictor of OCNPi. Since OCNPi is a diagnosis based on exclusion, the diagnosis can only be made by ensuring that there are no other explanatory causes. Currently, only a few studies have reported findings suggestive of OCNPi. One study group showed enhancement of the third cranial nerve on thin-section MRI but did not confirm the same findings of the fourth or sixth cranial nerves, and further validation is needed^18, 19^. WMH was associated with OCNPi regardless of the cranial nerve involved in this study.

The definition of the OCNPi varies between studies. One study defined presumed microvascular OCNP as a case in which symptoms improved within 6 months without other explainable causes. Another study defined an OCNPi as a case without other explainable causes and with at least one vascular risk factor. In this study, an OCNPi was defined as a case in which symptoms improved within 12 months without other explainable causes, regardless of the presence of vascular risk factors. These criteria were set because it is known that up to 85% of the OCNPi completely resolve within 12 months and typically resolve within 3 months^6, 7^. This reduced the number of patients with idiopathic OCNP (0.8%, 5/646) compared to those of other studies (2%–14%)^3, 7^.

In this study, 76 of 473 OCNPi patients (16.1%) and 68 of 173 OCNPo patients (39.3%) did not have vascular risk factors (i.e., hypertension, diabetes, dyslipidemia, or smoking). In a sensitivity analysis of this subgroup, both mild (grades 1–3) and moderate-to-severe (grades 4–6) WMH remained significant. This suggests that there is a common personal vulnerability for WMH and OCNPi other than the contribution of conventional vascular risk factors. Furthermore, we propose that a substantial proportion of patients previously diagnosed with idiopathic OCNP develop OCNP due to microvascular ischemia.

WMH, silent infarctions, and CMBs are representative biomarkers of cerebral SVD. These imaging findings refer to lesions mainly at the perforating arterial and arteriolar levels such as arteriosclerosis, lipohyalinosis, and fibrinoid necrosis. Cerebral SVDs can be observed in healthy elderly people as well as in individuals with vascular risk factors such as hypertension and smoking^20^. While silent infarction and CMBs are mainly due to a single perforating arterial or arteriolar occlusion or rupture, WMH is more diffusely distributed and observable from the early stage of SVD^10^. In addition to cerebral arteriosclerosis, blood–brain barrier dysfunction characterized by ischemic demyelination, gliosis, and selective neuronal necrosis at the capillary level increases the WMH burden^10, 21^. Similar to WMH, OCNPi shows pathological changes at both the arteriolar and capillary levels. Post-mortem pathology studies of the ischemic oculomotor nerve revealed arteriolar hyalinization and blood-nerve barrier dysfunction, characterized by edema and hypoxia of the endoneurial space^9, 22, 23^. Altogether, the association between WMH and OCNPi, not silent infarction or CMBs, among biomarkers of cerebral SVD suggests that a common pathophysiology of neurovascular dysfunction associated with both WMH and OCNPi might exist.

A greater proportion of OCNPi patients were taking antithrombotics at diagnosis compared with other OCNP patients in this study. This may be due to the more prevalent underlying diseases in patients with OCNPi. Whether antiplatelet agents could prevent OCNPi has not been elucidated. A study comparing OCNPi events between groups taking and not taking antiplatelet agents is needed to clarify this finding.

This study has some limitations. First, this was a medical record-based retrospective study conducted in two hospitals in South Korea. A prospective study is required to confirm the association between WMH and OCNPi. Second, inherent errors can exist in diagnosing OCNPi, because there is no definitive diagnostic test for OCNPi. All enrolled patients underwent MRI, which had a much wider range than the guidelines suggested: patients aged < 50 years, with history of any type of cancer at any time, with other neurologic signs or symptoms, with pupil-involving or partial third nerve palsy, and has no resolution 3 months after the initial visit^24^. Owing to the characteristics of the tertiary hospital, we performed additional workups, such as cerebrospinal fluid examination, if needed. Furthermore, the mean follow-up duration of 4.8 years after OCNP was sufficient to observe the patients’ symptoms and change the diagnosis as necessary. However, no direct pathological or neuroimaging evidence has been found. We cannot exclude other possible etiologies such as minor inflammation or demyelination. Since there are only three clinicopathological studies of OCNPi to date, more pathological or imaging studies are needed. Third, due to the heterogeneity of MRI scanners in our hospitals, MR images were obtained with heterogeneous acquisition parameters, although the proportion of 3 T MRI and SWI sequences was comparable between OCNPi and OCNPo.

In conclusion, we demonstrated that WMH is associated with ischemic isolated unilateral OCNP in patients aged ≥ 50 years. Owing to the lack of confirmatory diagnostic tests for OCNPi, the presence of WMH could predict OCNPi at early stage of admission.

## Supplementary Information

Supplementary Information.

## Data Availability

Data supporting the findings of this study are available from the corresponding author upon reasonable request.
