# Supplementary material for: Cerebral small vessel disease as imaging biomarker predicting ocular cranial nerve palsy of presumed ischemic origin at admission
Source: Sci Rep. 2022 Jul 18;12:12251. doi: 10.1038/s41598-022-16413-x (PMC9293888; doi:10.1038/s41598-022-16413-x)
Supplement: Supplementary file 1 — Supplementary Information. [file 41598_2022_16413_MOESM1_ESM.pdf]

Supplementary Information

Cerebral Small Vessel Disease as Imaging Biomarker Predicting Ocular Cranial Nerve Palsy of Presumed Ischemic Origin at Admission

Dong-Wan Kang[1], Sue Young Ha[1], Jung-Joon Sung[1], Hyunwoo Nam[2,*]

1. Department of Neurology, Seoul National University Hospital and Seoul National University College of Medicine, 101, Daehak-ro, Jongno-gu, Seoul, 03080, Republic of Korea

2. Department of Neurology, Seoul Metropolitan Government-Seoul National University Boramae Medical Center and Seoul National University College of Medicine, 20, Boramae-ro 5-gil, Dongjak-gu, Seoul, 07061, Republic of Korea

***Correspondence**

Hyunwoo Nam, MD, PhD

Seoul Metropolitan Government-Seoul National University Boramae Medical Center

20, Boramae-ro 5-gil, Dongjak-gu, Seoul, 07061, Republic of Korea

Tel: +82-2-870-2471; Fax: +82-2-831-3866

Email: hwnam85@gmail.com

MRI scanner (n)	Str (T)	Seq	FOV (mm)	TR (ms)	TE (ms)	Thk (mm)	FA (º)	Gap (mm)	IPR (mm)
**Philips Achieva (n = 56)**	1.5	FLAIR	240×240	11000	120	5	90	1.5	0.5×0.5
		GRE	240×240	810.1	25	5	25	2	0.5×0.5
**GE Healthcare SIGNA (n = 199)**	1.5	FLAIR	220×220	8802	124.2	5	90	1	0.5×0.5
		GRE	220×220	800	25	5	20	1	0.5×0.5
		SWI	240×240	59	5.9	3	20	0	0.5×0.5
**Siemens Sonata (n = 38)**	1.5	FLAIR	220×220	9310	130	5	150	1	0.7×0.7
		GRE	220×220	790	25	5	20	1	0.7×0.7
**Philips Achieva (n = 78)**	3	FLAIR	240×240	11000	120	5	90	1.5	0.5×0.5
		GRE	240×240	717.6	16.1	5	18	2	0.5×0.5
**Philips MRI Ingenia Elition (n = 45)**	3	FLAIR	220×220	9000	110	5	90	1	0.4×0.4
		SWI	220×220	30	0	2	17	0	0.4×0.4
**GE Healthcare Discovery MR750w (n = 55)**	3	FLAIR	220×220	9200	126.4	5	160	1	0.4×0.4
		SWI	229×229	37.1	23.2	4	25	-2	0.4×0.4
**GE Healthcare SIGNA (n = 27)**	3	FLAIR	220×220	9902	163.2	5	90	1	0.4×0.4
		GRE	220×220	467	25	5	20	1	0.4×0.4
**Siemens MAGNETOM Skyra (n = 44)**	3	FLAIR	220×220	8000	90	5	150	1	0.6×0.6
		SWI	199×219	29	20	2.5	17	0	0.5×0.5
**Siemens MAGNETOM Verio (n = 46)**	3	FLAIR	187×240	9000	97	5	140	1	0.6×0.6
		SWI	179×239	28	20	3	15	0	0.5×0.5
**Siemens MAGNETOM Vision Plus (n = 25)**	1.5	FLAIR	240×240	9000	119	5	180	2	0.9×0.9
		GRE	240×240	680	26	5	30	2	0.9×0.9
**Siemens MAGNETOM Impact Expert (n = 16)**	1.5	FLAIR	220×220	9000	119	6	180	1.2	0.9×0.9
		GRE	220×220	640	26	6	20	2	0.9×0.9
**Siemens MAGNETOM Avanto (n = 7)**	1.5	FLAIR	199×220	9000	98	5	150	1	0.4×0.4
		GRE	217×240	430	26	5	20	2	0.5×0.5
		SWI	199×220	52	40	2.5	15	0	0.6×0.6
**Siemens MAGNETOM Espree (n = 3)**	1.5	FLAIR	176×220	7530	125	5	150	1	0.7×0.7
		GRE	178×220	939	26	5	20	1	0.4×0.4
**Philips MRI Ingenia (n = 2)**	1.5	FLAIR	220×220	9000	110	5	90	1	0.4×0.4
		SWI	220×220	52	0	2	20	0	0.4×0.4
**Siemens MAGNETOM Symphony (n = 2)**	1.5	FLAIR	178×220	9000	115	5	150	2	0.4×0.4
		GRE	178×220	800	26	5	20	2	0.4×0.4

**Siemens MAGNETOM trio A Tim System (n = 3)**	3	FLAIR	199×220	9000	97	5	140	1	0.6×0.6
		SWI	178×220	28	20	2.5	15	0	0.5×0.5

Supplementary Table S1. MRI scanners and imaging acquisition parameters of fluid attenuated inversion recovery (FLAIR), T2*-weighted gradient echo (GRE), and susceptibility weighted images (SWI) were used in this study. Str, field strength; Seq, imaging sequence; FOV, field-of-view; TR, repetition time; TE, echo time; Thk, slice thickness; FA, flip angle; Gap, intersection gap.

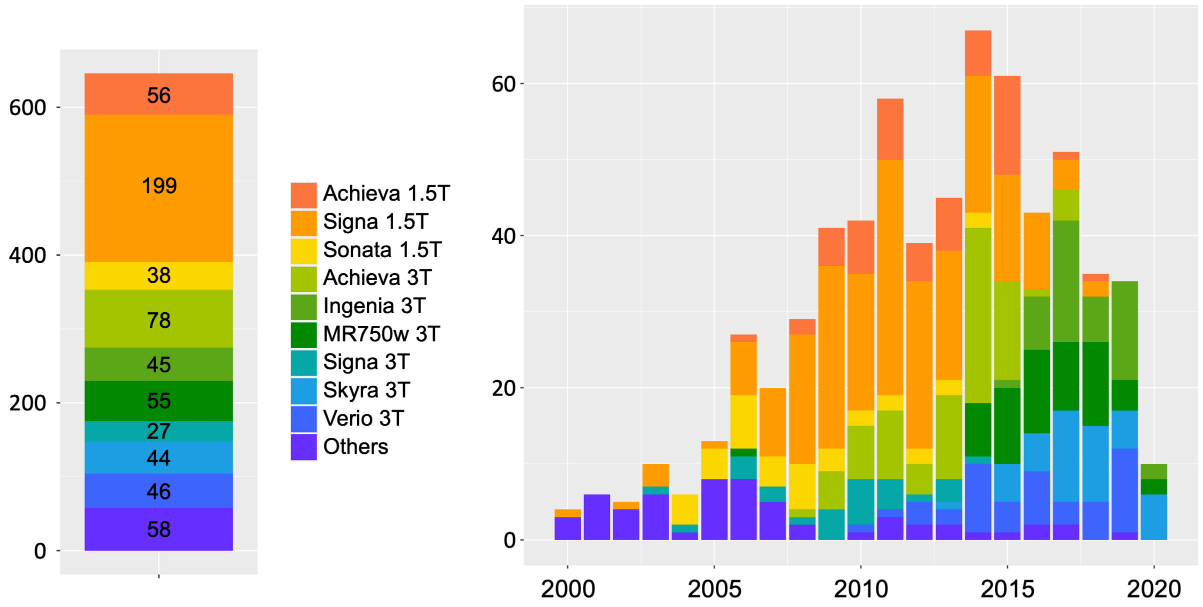

Supplementary Figure S1. Distribution of MRI scanners used by year.

	Total (N=646)
**WMH**	512 (79.3%)
0	134 (20.7%)
1	162 (25.1%)
2	199 (30.8%)
3	48 (7.4%)
4	65 (10.1%)
5	21 (3.3%)
6	17 (2.6%)
**PVWMH**	374 (57.9%)
0	272 (42.1%)
1	219 (33.9%)
2	98 (15.2%)
3	57 (8.8%)
**DWMH**	477 (73.8%)
0	169 (26.2%)
1	387 (59.9%)
2	72 (11.1%)
3	18 (2.8%)

Supplementary Table S2. Detailed description of baseline white matter hyperintensities (WMH), periventricular white matter hyperintensities (PVWMH), and deep white matter hyperintensities (DWMH).

Etiology	CN III (n, %)	CN IV (n, %)	CN VI (n, %)	Total (n, %)
**Presumed ischemic origin**	159 (62.4)	123 (88.5)	191 (75.8)	473 (73.2)
**Aneurysm**	25 (9.8)	0 (0)	6 (2.4)	31 (4.8)
**Cavernous sinus vascular lesions**	12 (4.7)	0 (0)	9 (3.6)	21 (3.3)
**Infection**	3 (1.2)	0 (0)	2 (0.8)	5 (0.8)
**Metabolic disorders**	0 (0)	0 (0)	1 (0.4)	1 (0.2)
**Inflammation**	20 (7.8)	2 (1.4)	13 (5.2)	35 (5.4)
**Autoimmune**	16 (6.3)	6 (4.3)	13 (5.2)	35 (5.4)
**Neoplasm**	17 (6.7)	7 (5.0)	15 (6.0)	39 (6.0)
**Others**	3 (1.2)	1 (0.7)	2 (0.8)	6 (0.9)

Supplementary Table S3. Etiologies of the patients with oculomotor (CN III), trochlear (CN IV), and abducens (CN VI) nerves.

	1.5T (N=348)	3T (N=298)	*P*-value*
**Male sex**	211 (60.6%)	187 (62.8%)	0.638
**Age**	66.2 ± 8.8	67.3 ± 8.6	0.126
**BMI (kg/m^2)**	24.4 ± 3.4	24.4 ± 3.7	0.844
**Diagnosis**			0.43
CN III	144 (41.4%)	111 (37.2%)	
CN IV	76 (21.8%)	63 (21.1%)	
CN VI	128 (36.8%)	124 (41.6%)	
**OCNPi**	260 (74.7%)	213 (71.5%)	0.403
**Periorbital pain or headache**	163 (46.8%)	121 (40.6%)	0.131
**WMH**			0.394
No (0)	77 (22.1%)	57 (19.1%)	
Mild (1–3)	212 (60.9%)	197 (66.1%)	
Moderate to Severe (4–6)	59 (17.0%)	44 (14.8%)	
**PVWMH**			0.044
0	157 (45.1%)	115 (38.6%)	
Mild (1)	103 (29.6%)	116 (38.9%)	
Moderate to severe (2–3)	88 (25.3%)	67 (22.5%)	
**DWMH**			0.852
0	93 (26.7%)	76 (25.5%)	
Mild (1)	205 (58.9%)	182 (61.1%)	
Moderate to severe (2–3)	50 (14.4%)	40 (13.4%)	
**Silent infarction**	83 (23.9%)	69 (23.2%)	0.927
**CMBs**	39 (12.4%)	47 (18.4%)	0.061
**Vascular risk factors**	265 (76.1%)	237 (79.5%)	0.35
**Hypertension**	178 (51.1%)	163 (54.7%)	0.411
**Diabetes**	148 (42.5%)	128 (43.0%)	0.977
**Dyslipidemia**	83 (23.9%)	91 (30.5%)	0.069
**Smoking**	59 (17.0%)	39 (13.1%)	0.209
**Ischemic heart disease**	38 (10.9%)	24 (8.1%)	0.272
**History of stroke**	21 (6.0%)	12 (4.0%)	0.329
**HbA1c (%)**	6.7 ± 1.5	6.7 ± 1.6	0.741
**Chronic kidney disease**	46 (13.3%)	34 (11.4%)	0.555
**eGFR (mL/min/1.73m^2)**	80.4 ± 24.5	83.8 ± 25.1	0.084
**Prior use of antithrombotics**	69 (19.9%)	57 (19.2%)	0.903
**Follow up duration after OCNP (yr)**	5.8 ± 4.3	3.5 ± 3.1	<0.001

Supplementary Table S4. Comparison of clinical and imaging parameters according to the MRI field strength. BMI, body mass index; CN, cranial nerve; OCNPi, ocular cranial nerve palsy of presumed ischemic origin; WMH, white matter hyperintensities; PVWMH, periventricular white matter hyperintensities; DWMH, deep white matter hyperintensities; CMBs, cerebral microbleeds; eGFR, estimated glomerular filtration rate; OCNP, ocular cranial nerve palsy. Vascular risk factors refer to the presence of at least one of the following: hypertension, diabetes,

dyslipidemia, and/or smoking. *Student's t-test for continuous variables and chi-square test for categorical variables.

	GRE (N = 327)	SWI (N = 244)	*P*-value*
**Male sex**	188 (57.5%)	157 (64.3%)	0.116
**Age**	66.5 ± 8.7	67.3 ± 8.6	0.308
**BMI (kg/m^2)**	24.4 ± 3.6	24.3 ± 3.4	0.704
**Diagnosis**			0.94
CN III	128 (39.1%)	92 (37.7%)	
CN IV	69 (21.1%)	53 (21.7%)	
CN VI	130 (39.8%)	99 (40.6%)	
**OCNPi**	237 (72.5%)	181 (74.2%)	0.72
**Periorbital pain or headache**	157 (48.0%)	121 (49.6%)	0.773
**MRI field strength (T)**			<0.001
1.5T	236 (72.2%)	80 (32.8%)	
3T	91 (27.8%)	164 (67.2%)	
**WMH**			0.462
No (0)	69 (21.1%)	52 (21.3%)	
Mild (1–3)	200 (61.2%)	158 (64.8%)	
Moderate to Severe (4–6)	58 (17.7%)	34 (13.9%)	
**PVWMH**			0.101
0	147 (45.0%)	96 (39.3%)	
Mild (1)	98 (30.0%)	94 (38.5%)	
Moderate to severe (2–3)	82 (25.1%)	54 (22.1%)	
**DWMH**			0.473
0	81 (24.8%)	68 (27.9%)	
Mild (1)	194 (59.3%)	145 (59.4%)	
Moderate to severe (2–3)	52 (15.9%)	31 (12.7%)	
**Silent infarction**	75 (22.9%)	58 (23.8%)	0.894
**CMBs**	37 (11.3%)	49 (20.2%)	0.005
**Vascular risk factors**	261 (79.8%)	187 (76.6%)	0.418
**Hypertension**	183 (56.0%)	122 (50.0%)	0.184
**Diabetes**	146 (44.6%)	100 (41.0%)	0.43
**Dyslipidemia**	85 (26.0%)	73 (29.9%)	0.346
**Smoking**	59 (18.0%)	29 (11.9%)	0.058
**Ischemic heart disease**	29 (8.9%)	29 (11.9%)	0.298
**History of stroke**	21 (6.4%)	10 (4.1%)	0.305
**HbA1c (%)**	6.8 ± 1.5	6.6 ± 1.4	0.203
**Chronic kidney disease**	47 (14.4%)	23 (9.5%)	0.102
**eGFR (mL/min/1.73m^2)**	79.8 ± 24.5	84.8 ± 25.6	0.019
**Prior use of antithrombotics**	67 (20.5%)	48 (19.8%)	0.931
**Follow up duration after OCNP (yr)**	5.7 ± 4.3	2.8 ± 2.4	<0.001

Supplementary Table S5. Comparison of clinical and imaging parameters according to susceptibility sequence type GRE, T2*-weighted gradient echo; SWI, susceptibility weighted image; BMI, body mass index; CN, cranial nerve; OCNPi, ocular cranial nerve palsy of presumed ischemic origin; WMH, white matter hyperintensities;

PVWMH, periventricular white matter hyperintensities; DWMH, deep white matter hyperintensities; CMBs, cerebral microbleeds; eGFR, estimated glomerular filtration rate; OCNP, ocular cranial nerve palsy. Vascular risk factors refer to the presence of at least one of the following: hypertension, diabetes, dyslipidemia, and/or smoking.
*Student's t-test for continuous variables and chi-square test for categorical variables.

	OCNPi	
	**Odds ratio (95% C.I.)**	***P*-value**
**Model 4.** PVWMH		
No (0)	Reference	Reference
Mild (1)	1.61 (0.92–2.81)	0.093
Moderate to severe (2–3)	1.48 (0.73–3.01)	0.275
**Model 5.** DWMH		
No (0)	Reference	Reference
Mild (1)	2.26 (1.31–3.91)	0.004
Moderate to severe (2–3)	1.66 (0.72–3.82)	0.233

Supplementary Table S6. Odds ratios of imaging parameters based on multivariate logistic regression analysis. In each model, the covariates were age, sex, diagnosis, MRI field strength (3T vs. 1.5T), body mass index, hypertension, diabetes, ischemic heart disease, estimated glomerular filtration rate, prior use of antithrombotics, and dyslipidemia. OCNPi, ocular cranial nerve palsy of presumed ischemic origin; PVWMH, periventricular white matter hyperintensities; DWMH, deep white matter hyperintensities.

	Total (N = 144)	OCNPi (N = 76)	OCNPo (N = 68)	*P*-value*
**Male sex**	76 (52.8%)	50 (65.8%)	26 (38.2%)	0.002
**Age**	65.0 ± 9.2	66.1 ± 8.9	63.7 ± 9.4	0.114
**BMI (kg/m^2)**	23.3 ± 3.5	23.6 ± 3.1	23.0 ± 3.7	0.354
**Diagnosis**				0.001
CN III	56 (38.9%)	21 (27.6%)	35 (51.5%)	
CN IV	35 (24.3%)	27 (35.5%)	8 (11.8%)	
CN VI	53 (36.8%)	28 (36.8%)	25 (36.8%)	
**WMH**				0.033
No (0)	38 (26.4%)	14 (18.4%)	24 (35.3%)	
Mild (1–3)	91 (63.2%)	51 (67.1%)	40 (58.8%)	
Moderate to Severe (4–6)	15 (10.4%)	11 (14.5%)	4 (5.9%)	
**PVWMH**				0.136
0	76 (52.8%)	35 (46.1%)	41 (60.3%)	
Mild (1)	48 (33.3%)	27 (35.5%)	21 (30.9%)	
Moderate to severe (2–3)	20 (13.9%)	14 (18.4%)	6 (8.8%)	
**DWMH**				0.057
0	44 (30.6%)	18 (23.7%)	26 (38.2%)	
Mild (1)	87 (60.4%)	48 (63.2%)	39 (57.4%)	
Moderate to severe (2–3)	13 (9.0%)	10 (13.2%)	3 (4.4%)	
**Silent infarction**	20 (14.0%)	13 (17.3%)	7 (10.3%)	0.332
**CMBs**	17 (13.9%)	11 (16.9%)	6 (10.5%)	0.45
**MRI field strength**				0.436
1.5T	83 (57.6%)	41 (53.9%)	42 (61.8%)	
3T	61 (42.4%)	35 (46.1%)	26 (38.2%)	
**Susceptibility sequence**				0.618
GRE	66 (53.7%)	33 (50.8%)	33 (56.9%)	
SWI	57 (46.3%)	32 (49.2%)	25 (43.1%)	
**Ischemic heart disease**	6 (4.2%)	4 (5.3%)	2 (2.9%)	0.781
**History of stroke**	2 (1.4%)	2 (2.6%)	0 (0.0%)	0.526
**HbA1c (%)**	5.6 ± 0.4	5.6 ± 0.5	5.6 ± 0.3	0.791
**Chronic kidney disease**	9 (6.3%)	4 (5.3%)	5 (7.4%)	0.879
**eGFR (mL/min/1.73m^2)**	86.5 ± 26.5	84.8 ± 23.7	88.4 ± 29.4	0.432
**Prior use of antithrombotics**	6 (4.2%)	4 (5.3%)	2 (3.0%)	0.795
**Follow up duration after OCNP (yr)**	4.8 ± 4.1	5.8 ± 4.2	4.4 ± 4.1	0.141

Supplementary Table S7. Comparison of ocular cranial nerve palsy of presumed ischemic origin (OCNPi) with ocular cranial nerve palsy of other origin (OCNPo) among patients without vascular risk factors. BMI, body mass index; CN, cranial nerve; OCNP, ocular cranial nerve palsy; WMH, white matter hyperintensities; PVWMH, periventricular white matter hyperintensities; DWMH, deep white matter hyperintensities; CMBs, cerebral microbleeds; GRE, T2*-weighted gradient echo; SWI, susceptibility-weighted image; eGFR, estimated glomerular filtration rate. Vascular risk factors refer to the presence of at least one of the following: hypertension,

diabetes, dyslipidemia, and/or smoking. *Student's t-test for continuous variables and chi-square test for categorical variables.
